# Phenylephrine Usage During Anesthesia in Concussed Patients Undergoing Orthopedic Surgery: A Retrospective Cohort Study

**DOI:** 10.7759/cureus.79046

**Published:** 2025-02-15

**Authors:** Jonathan Henning, Brian Villa, Parker Penny, Trevor Lin, Jose J Diaz, Jeffrey Weiss, John Hodgson, Enrico M Camporesi

**Affiliations:** 1 Anesthesiology, University of South Florida Morsani College of Medicine, Tampa, USA; 2 Acute Care Surgery, University of South Florida Morsani College of Medicine, Tampa General Hospital, Tampa, USA; 3 Anesthesiology and Perioperative Medicine, University of South Florida Morsani College of Medicine, Tampa General Hospital, Tampa, USA; 4 Anesthesiology, University of South Florida Morsani College of Medicine, Tampa General Hospital, Tampa, USA; 5 Surgery, University of South Florida Morsani College of Medicine, Tampa General Hospital, Tampa, USA

**Keywords:** anesthesia and concussion, concussion, loss of consciousness, mild traumatic brain injury, surgical care for concussed patients

## Abstract

Background and objective

Concussion can lead to complex physiologic changes, including alterations to cerebral blow flow autoregulation. Based on this understanding, we aimed to analyze if concussed patients undergoing orthopedic surgery required a higher dosage or a more frequent administration of phenylephrine while under general anesthesia; we also sought to assess if a concussion with an associated loss of consciousness (LOC) influenced treatment.

Methods

We performed a retrospective review of the data of patients admitted in the past three years to the emergency room (ER) with a diagnosis of head concussion at our level-one trauma center. Patients were filtered by selecting those who underwent a single emergent orthopedic surgical procedure under general anesthesia: a total of 61 individuals. We further refined this group according to the presence/absence of LOC. The cohort with concussion coupled with LOC included 50 patients (82% of the treatment group), and the cohort with concussion without LOC had 11 patients (18% of the treatment group). Concussed patients were compared to a matched group of 118 patients without head trauma who also presented to the ER in the same period and required similar emergent orthopedic surgeries during their hospitalization. Phenylephrine was the most common vasopressor used in all surgeries. Statistical analyses involved chi-square tests for the frequency of phenylephrine usage. The Kruskal-Wallis and Dunn's tests were also used to examine differences in phenylephrine dosage across each cohort.

Results

In all concussed patients, regardless of LOC, there was no difference in phenylephrine usage during general anesthesia compared to controls without head trauma. However, concussed patients who experienced LOC required significantly higher doses of phenylephrine compared to controls.

Conclusions

This is the first study to explore the relationship between concussion, LOC, and vasopressor usage during general anesthesia. Its findings demonstrated an increased requirement for phenylephrine in concussed patients with LOC compared to controls.

## Introduction

Concussions refer to a specific type of traumatic brain injury (TBI) defined as a traumatically induced disturbance of brain function. TBIs vary on the scale of their severity, with concussions usually being associated with mild traumatic brain injury (mTBI). Concussions, although a subset of mTBI, are often used to describe any type of TBI [1]. However, concussions are technically described as a mTBI without structural abnormalities [2,3]. Concussions are extremely common, and their estimated incidence in the United States ranges from 1.4 to 3.8 million cases each year [1]. Sports-related concussions account for the majority of cases, and these are especially a cause of concern in children, adolescents, and young adults [4]. Commonly reported symptoms of concussions include headache, dizziness, and fatigue [4,5]. The duration of symptoms from concussions varies, and excluding some exceptions, symptoms resolve within two weeks in most patients [4-6].

Numerous physiological changes may occur following a concussion. A notable post-concussion physiologic change entails a disruption to cerebral blood flow autoregulation [7,8]. Based on this potential disruption of vascular tone, we investigated if concussed patients required a higher phenylephrine dosage or more frequent administration during general anesthesia compared to a control group of non-concussed patients. In addition, we examined if the presence of a loss of consciousness (LOC) resulting from the head trauma had any effects on phenylephrine use. We hypothesized that concussed patients would have an increased phenylephrine requirement, and we also expected concussion with LOC to be associated with even greater phenylephrine requirements.

## Materials and methods

This study was carried out in the Department of Anesthesiology and Perioperative Medicine, University of South Florida Morsani College of Medicine, Tampa, Florida, United States. The study was approved by the Institutional Review Board of the University of South Florida. Data of patients who were seen from January 2020 to September 2023 were extracted from the electronic medical records at our level-one trauma center. Data of patients admitted with a confirmed diagnosis of concussion (based on the ICD-10 code from the trauma admission) during this period were identified and retrospectively reviewed.

From the identified pool of patients with concussions, those who underwent orthopedic surgeries requiring general anesthesia during their hospitalization were selected for inclusion in the study. Only those patients diagnosed with concussions were included in the treatment group. Patients with all other types of head trauma, as well as patients with findings on head CT, were excluded. Patients with unspecified LOC and those requiring multiple surgeries during their hospitalization were also excluded. The Injury Severity Score (ISS) and Glasgow Coma Scale (GCS) scores were cataloged for each concussed patient, and the breakdown of these scores is detailed in the Results section. Patients with a GCS score of less than 13 were excluded since this is the expected range for a diagnosis of mTBI [9]. The distribution of injury severity according to ISS was as follows: minor (1-8), moderate (9-15), severe (16-24), and very severe (≥25) injuries; there were multiple cases of severe and very severe injuries.

Many patients required surgical repairs at different anatomical sites during the same operation, and they were excluded as we focused on patients who only underwent one specific surgery; these procedures encompassed repairs to the humerus, radius, ulna, pelvis, femur, tibia, fibula, and ankle. Patients with spinal orthopedic procedures were excluded since they nearly always require a continuous phenylephrine infusion as well as alterations in the anesthetic plan to accommodate neurological monitoring. The treatment group comprised 61 concussed patients who met the criteria of having both a diagnosed concussion and undergone a single isolated orthopedic surgical procedure under general anesthesia. This group was further classified, adding a subgroup involving concussion with LOC. The cohort with concussion with LOC included 50 patients (82% of the treatment group), and the cohort with concussion without LOC had 11 patients (18% of the treatment group); for patients with documented LOC, the duration of LOC was brief, reportedly not more than five minutes for any patient.

To create a comparative cohort, patients with no head trauma (no concussion or LOC) who also presented to the emergency room (ER) in the same period and required similar emergent orthopedic surgeries during their hospitalization were identified. This control group totaled 118 patients. The selection process for this group involved matching the concussed patients based on specific criteria: sex, age (±2 years), and specific orthopedic procedure performed. We achieved an approximate 1:2 matching ratio for concussed patients (regardless of LOC) to those with no head trauma. Regarding demographics, among the control group (n=118), 32 patients (27%) were female, and 86 (73%) were male. In the concussion with LOC group (n=50), there were 14 females (28%) and 36 males (72%). In the concussion without LOC group (n=11), seven participants (64%) were female, and four (36%) were male. Age demographic data are detailed in the Results section. 

The details of all vasopressors used during surgery were documented, and the most common vasopressor was phenylephrine. Only a minority of patients received additional or alternative vasopressors, such as ephedrine. There was no observable difference in the usage of alternative vasopressors between the groups. The total dosage of phenylephrine required during anesthesia was recorded for each patient. The intraoperative decision to administer phenylephrine was made based on the clinical judgment of each anesthesia provider, a combination of anesthesiologists and certified registered nurse anesthetists. 

Of note, the blood pressures of all patients were monitored using a noninvasive blood pressure cuff. In addition, 10 patients (16%) from the concussed cohort and eight (7%) from the non-head trauma cohort had arterial lines for continuous invasive blood pressure measurement. Estimated blood loss for all patients was documented, and none of the patients required blood transfusions. Transfusions were to be initiated per institutional protocol if hemoglobin dropped below the threshold of 7 g/dL or if there was clinical evidence of substantial blood loss and hemodynamic compromise. Sevoflurane was used to maintain anesthesia in all but three patients. Certain cases also received nitrous oxide, propofol, or dexmedetomidine as part of the maintenance regimen. Of the three patients not on inhalational anesthesia, each received maintenance with propofol; only one of these patients received phenylephrine.

We used the chi-square test to examine the relationship between the frequency of phenylephrine use during general anesthesia and the concussed vs. non-concussed groups. We employed the Mann-Whitney U test to compare age and total phenylephrine dosage between the concussion and non-concussed groups. We applied the Kruskal-Wallis test followed by post-hoc Dunn's test to examine differences in length of stay and phenylephrine dosage across three cohorts: concussed with LOC, concussed without LOC, and no head trauma. A p-value of less than 0.05 was considered statistically significant for all analyses.

## Results

Table 1 presents the details of ISS and GCS scores of concussion patients classified based on LOC status.

**Table 1:** Breakdown of the ISS and GCS scores for the concussed patients, categorized by LOC status The GCS of one patient in the concussion with LOC cohort was not found in the medical record GCS: Glasgow Coma Scale; ISS: Injury Severity Score; LOC: loss of consciousness

Cohort	ISS	N (%)	GCS score	N (%)
Concussion with LOC (n=50)	4	1 (2%)	13	2 (4%)
	8	5 (10%)		
	9	10 (20%)		
	11	1 (2%)		
	12	3 (6%)	14	7 (4%)
	13	1 (2%)		
	14	11 (22%)		
	17	9 (18%)		
	21	1 (2%)	15	40 (80%)
	22	4 (8%)		
	24	2 (4%)		
	29	2 (4%)		
Concussion without LOC (n=11)	9	3 (27%)	14	1 (9%)
	12	1 (9%)		
	14	4 (36%)		
	17	1 (9%)	15	10 (91%)
	22	1 (9%)		
	29	1 (9%)		

Table 2 summarizes the distribution of the anatomical location of orthopedic procedures.

**Table 2:** Distribution of the anatomical location of orthopedic procedures in various groups The most common procedure was open-reduction internal fixation at each site LOC: loss of consciousness

Procedure location	Concussed with LOC group (n=50), n (%)	Concussed without LOC group (n=11), n (%)	Control group (n=118)
Pelvic	8 (16%)	1 (9%)	16 (14%)
Femur	13 (26%)	2 (18%)	30 (25%)
Tibia and/or fibula	12 (24%)	4 (36%)	32 (27%)
Ankle	7 (14%)	2 (18%)	18 (15%)
Humerus	3 (6%)	0 (0%)	6 (5%)
Radius and ulna	7 (14%)	2 (18%)	16 (14%)

Table 3 provides the age-group-wide distribution of patients in various groups. 

**Table 3:** Breakdown of age-related demographics across concussed and control groups The age-related comparison between the concussed and control groups revealed no statistically significant difference (Mann-Whitney U, p=0.607). Age comparison across these groups was not significantly different when considering LOC status (Kruskal-Wallis, p=0.858). A p-value of <0.05 was considered statistically significant LOC: loss of consciousness

Cohort	Age group, years	N (%)
Concussion with LOC (n=50)	1-9	3 (6%)
	10-19	19 (38%)
	20-29	7 (14%)
	30-39	8 (16%)
	40-49	5 (10%)
	50-59	3 (6%)
	60-69	2 (4%)
	70-79	2 (4%)
	80-89	1 (2%)
Concussion without LOC (n=11)	1-9	1 (9%)
	10-19	4 (36%)
	20-29	2 (18%)
	30-39	0 (0%)
	40-49	1 (9%)
	50-59	0 (0%)
	60-69	3 (27%)
	70-79	0 (0%)
	80-89	0 (0%)
Control (n=118)	1-9	4 (3%)
	10-19	46 (39%)
	20-29	16 (14%)
	30-39	23 (19%)
	40-49	6 (5%)
	50-59	9 (8%)
	60-69	8 (7%)
	70-79	6 (5%)
	80-89	0 (0%)

Our first analysis involved comparing concussed patients to the control group regardless of LOC status. Regarding phenylephrine dosage, the mean dosage in patients who received phenylephrine in the control was 312.24 ± 279.23 mcg, while it was 513.10 ± 497.93 mcg in the concussed group. The Mann-Whitney U test, conducted using data from the patients who received phenylephrine, indicated no statistically significant differences in phenylephrine dosages between the concussed and non-concussed groups (p=0.073). Similarly, chi-square tests analyzing the relationship between the frequency of phenylephrine use and the categorization of patients into treatment or control groups showed no statistically significant differences (p=0.121).

In the comparison of phenylephrine usage between the concussed and no-head trauma patients, we also included LOC status, leading to statistically significant findings. This analysis involved three groups: +concussion/+LOC, +concussion/-LOC, and -concussion/-LOC. Age comparison across these groups did not show any significantly different difference (Kruskal Wallis, p=0.858). Chi-square tests found significant differences in the sex distribution of the +concussion/-LOC group compared to the other groups due to the reduced size of this group (Chi-square, p=0.024 and p=0.012 when +concussion/-LOC is compared to +concussion/+LOC and control, respectively).

There were no significant differences in sex distribution between the -concussion/-LOC and +concussion/+LOC groups. The average length of stay in the hospital was 5.20 ± 4.98 days for the concussion with LOC cohort and 4.09 ± 2.30 days for the concussion without LOC cohort. The average length of stay in the hospital for the non-head trauma cohort was 2.73 ± 1.86 days. The length of hospital stay was significantly different across these groups (Kruskal Wallis, p=1.49×10-4), and Dunn's test showed that the +concussion/+LOC and +concussion/-LOC groups both had significantly longer lengths of stay than the no-head trauma group (p=8.39×10-5 and p=0.038).

The frequency of phenylephrine use was not significantly higher when comparing any of the groups: -concussion/-LOC vs. +concussion/-LOC (chi-square, p=0.959), -concussion/-LOC vs. +concussion/+LOC (chi-square, p=0.081), and +concussion/-LOC vs. +concussion/+LOC (chi-square, p=0.412). However, the mean dosages in patients who received phenylephrine differed significantly across the groups (Table 4). The Kruskal-Wallis test, also calculated among those who received phenylephrine, showed the dosages of phenylephrine used in these groups to be significantly different (p=0.043). Furthermore, the post-hoc Dunn's test specified that dosages were larger and statistically significant for concussed patients with LOC compared to patients without any head trauma (Table 4).

**Table 4:** Mean and median dosages of phenylephrine across each cohort *Administered in separate boluses Data presented as the mean ± standard deviation (SD) and median phenylephrine dosage (mcg) for each group. These dosages were statistically significantly different (p=0.043, Kruskal Wallis); Dunn's test showed that the phenylephrine dosages were significantly larger for the concussed patients with LOC when compared to patients without head trauma (p=0.025). A p-value of <0.05 was considered statistically significant LOC: loss of consciousness

Group	Mean phenylephrine dose (mcg) per case*	Median phenylephrine dose (mcg) per case*	Pair for comparison	z-score (Dunn's test)	P-value (Dunn's test)
-Concussion/-LOC	312.24 ± 279.23	200	-Concussion/-LOC vs. +Concussion/-LOC	0.749	0.454
+Concussion/-LOC	175.00 ± 95.74	150	-Concussion/-LOC vs. +Concussion/+LOC	2.241	0.025
+Concussion/+LOC	567.20 ± 497.93	400	+Concussion/-LOC vs. +Concussion/+LOC	1.745	0.081

## Discussion

Our results suggest that trauma patients with concussions who also suffered from LOC required significantly more phenylephrine during emergency surgery compared to the control group without concussions. The brain may undergo several complex pathophysiological shifts following a concussion, including metabolic alterations, ionic shifts, disruption to cerebral blood flow autoregulation, autonomic dysfunction, neurotransmitter release, disruption to the blood-brain barrier, traumatic axonal injury, pituitary dysfunction, and expression of inflammatory cytokines [7,10-12]. Based on the theory that the brain does not pressure autoregulate as it usually should after a concussion [10], we hypothesized that concussed patients undergoing anesthesia would require a higher dosage of vasopressors (phenylephrine). However, when not factoring in LOC in the analysis, our results did not support this hypothesis since we found no significant difference in the frequency of phenylephrine use or total phenylephrine dosages between concussed and non-concussed patients undergoing orthopedic surgery. The differences in mean dosage for the control group (312.24 ± 279.23 mcg) compared to the concussed group (513.10 ± 497.93 mcg) are worth acknowledging. However, a comparison via the Mann-Whitney U test showed these differences to be insignificant (p=0.073).

Separating the concussed cohort according to LOC status yielded intriguing findings. We found a statistically significant difference in phenylephrine dosages in concussed patients with LOC vs. patients with no head trauma. This result is only slightly different than our original hypothesis, where we anticipated finding higher usage of phenylephrine in all patients with concussions. Based on this association, LOC or higher severity of head trauma could be critical to the physiological changes that occur with concussions. Perhaps LOC signals severe enough head trauma for the changes in autoregulation to manifest. The literature on post-concussion physiology is inconclusive regarding the threshold of injury to trigger physiologic shifts [7]. There is a need for concussion research to specify differences in physiological changes for concussions with LOC compared to concussions without LOC. The association between concussion and phenylephrine usage could be important in the management of concussed patients by anesthesiologists. Though there are currently no guidelines for managing concussed patients undergoing general anesthesia, it is well-established that extra caution might be necessary when anesthetizing a patient with a concussed brain [7,13-17]. Despite widespread agreement, research on complications that may occur when a concussed patient receives general anesthesia is lacking.

Our literature review on complications associated with concussed patients undergoing anesthesia examined all types of articles, including case reports. Still, we only found one study that directly analyzed the perioperative complications in concussed patients undergoing general anesthesia compared to non-concussed patients. This study by D'Souza et al. found no differences in intraoperative and postoperative complications when comparing patients with a concussion to patients without a concussion [18]. Our results align with this as we also did not find significant differences between concussed and non-concussed patients undergoing anesthesia when LOC is not considered. Furthermore, our review of the records in the concussion group did not find any suboptimal outcomes. It should be noted that D'Souza et al. did not consider LOC in their analysis. Once we controlled for LOC, our analysis revealed statistically significant differences. Given that D'Souza et al.'s is the only study to investigate the complications associated with concussion and general anesthesia, and ours is the first to analyze vasopressor requirements for the concussed patient undergoing anesthesia, there is a considerable need for more research into the connection between concussion focusing on LOC and general anesthesia to validate these findings.

This study has a few limitations. A larger sample size of concussed patients may have revealed significant differences in the total vasoactive dose required for concussed and non-concussed patients, regardless of LOC status. Due to the retrospective nature of our study, the lack of a standardized threshold for phenylephrine administration is a notable limitation. Other limitations of this study include its single-institution design, which limits generalizability, and the exclusion of complex surgeries, which could introduce selection bias.

## Conclusions

The effects of general anesthesia among individuals with a history of concussion or LOC remains a pressing and inadequately addressed question. Post-concussion, the brain undergoes a complex array of physiologic changes that may present a risk for a second injury. Our investigation revealed statistically significant differences in total phenylephrine administered when considering concussed patients with an associated LOC. This cohort of concussed patients with LOC required a statistically significant increase in phenylephrine administration compared to patients without head trauma. This association may suggest that LOC may be a marker of significant physiologic changes that occur with a concussion, which may have an effect on treatment and eventual outcomes.

## References

[1] Ferry B, DeCastro A (2023). Concussion. https://www.ncbi.nlm.nih.gov/books/NBK537017/.

[2] Coenen J, Reinsberger C (2023). Neurophysiological markers to guide return to sport after sport-related concussion. J Clin Neurophysiol.

[3] Stephens JA, Liu P, Lu H, Suskauer SJ (2018). Cerebral blood flow after mild traumatic brain injury: associations between symptoms and post-injury perfusion. J Neurotrauma.

[4] Noble JM, Hesdorffer DC (2013). Sport-related concussions: a review of epidemiology, challenges in diagnosis, and potential risk factors. Neuropsychol Rev.

[5] Eisenberg MA, Meehan WP 3rd, Mannix R (2014). Duration and course of post-concussive symptoms. Pediatrics.

[6] McCrea M, Guskiewicz KM, Marshall SW (2003). Acute effects and recovery time following concussion in collegiate football players: the NCAA Concussion Study. JAMA.

[7] Rasouli MR, Kavin M, Stache S, Mahla ME, Schwenk ES (2020). Anesthesia for the patient with a recently diagnosed concussion: think about the brain!. Korean J Anesthesiol.

[8] Strebel S, Lam AM, Matta BF, Newell DW (1997). Impaired cerebral autoregulation after mild brain injury. Surg Neurol.

[9] Jain S, Iverson LM (2024). Glasgow Coma Scale. https://www.ncbi.nlm.nih.gov/books/NBK513298/.

[10] Choe MC (2016). The pathophysiology of concussion. Curr Pain Headache Rep.

[11] Giza CC, Hovda DA (2014). The new neurometabolic cascade of concussion. Neurosurgery.

[12] Barkhoudarian G, Hovda DA, Giza CC (2016). The molecular pathophysiology of concussive brain injury - an update. Phys Med Rehabil Clin N Am.

[13] Vavilala MS, Ferrari LR, Herring SA (2017). Perioperative care of the concussed patient: making the case for defining best anesthesia care. Anesth Analg.

[14] Pasternak JJ, Abcejo AS (2020). Anesthesia and the brain after concussion. Curr Opin Anaesthesiol.

[15] Tasker RC (2017). Anesthesia and concussion. Curr Opin Anaesthesiol.

[16] Abcejo AS, Savica R, Lanier WL, Pasternak JJ (2017). Exposure to surgery and anesthesia after concussion due to mild traumatic brain injury. Mayo Clin Proc.

[17] King D, Collins-Yoder A (2019). Perioperative considerations in patients with concussion. AANA J.

[18] D'Souza RS, Sexton MA, Schulte PJ, Pasternak JJ, Abcejo AS (2021). Recent preoperative concussion and postoperative complications: a retrospective matched-cohort study. J Neurosurg Anesthesiol.

